# Effects of Nitrogen Application Rate and Leaf Age on the Distribution Pattern of Leaf SPAD Readings in the Rice Canopy

**DOI:** 10.1371/journal.pone.0088421

**Published:** 2014-02-10

**Authors:** Hu Yang, Jinwen Li, Jingping Yang, Hua Wang, Junliang Zou, Junjun He

**Affiliations:** 1 Institute of Environmental Protection, College of Environmental and Resource Sciences, Zhejiang University, Hangzhou, China; 2 Shanghai Academy of Environmental Science, Shanghai, China; Tennessee State University, United States of America

## Abstract

A Soil-Plant Analysis Development (SPAD) chlorophyll meter can be used as a simple tool for evaluating N concentration of the leaf and investigating the combined effects of nitrogen rate and leaf age on N distribution. We conducted experiments in a paddy field over two consecutive years (2008–2009) using rice plants treated with six different N application levels. N distribution pattern was determined by SPAD readings based on the temporal dynamics of N concentrations in individual leaves. At 62 days after transplantation (DAT) in 2008 and DAT 60 in 2009, leaf SPAD readings increased from the upper to lower in the rice canopy that received N levels of 150 to 375 kg ha^−1^The differences in SPAD readings between the upper and lower leaf were larger under higher N application rates. However, as plants grew, this atypical distribution of SPAD readings in canopy leaf quickly reversed to the general order. In addition, temporal dynamics of the leaf SPAD readings (N concentrations) were fitted to a piecewise function. In our model, changes in leaf SPAD readings were divided into three stages: growth, functioning, and senescence periods. The leaf growth period lasted approximately 6 days, and cumulative growing days were not affected by N application rates. The leaf functioning period was represented with a relatively stable SPAD reading related to N application rate, and cumulative growing days were extended with increasing N application rates. A quadratic equation was utilized to describe the relationship between SPAD readings and leaf age during the leaf senescence period. The rate of decrease in SPAD readings increased with the age of leaves, but the rate was slowed by N application. As leaves in the lower canopy were physiologically older than leaves in the upper canopy, the rate of decrease in SPAD readings was faster in the lower leaves.

## Introduction

During plant canopy development, a photosynthetic photon flux density (PPFD) gradient is formed in which PPFD decreases with increasing canopy depth [1]. To optimize nitrogen (N) distribution in the canopy, carbon gain, especially in plants growing in dense stands, is maximized when a gradient of N concentration per unit area (*N*_a_) is established in parallel with the PPFD gradient [2]–[5]. The preferential distribution of N to the upper leaves in such a canopy, in contrast to a canopy with a uniform distribution, leads to higher rates of canopy photosynthesis [3]. The vertical *N*_a_ gradient is, in fact, regarded as an adaptive response of leaves to the light gradient inside the canopy. Leaf N distribution within the canopy can be described by the equation: 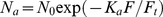
(1)


Where *F* is the cumulative leaf area index (LAI) from the top of the canopy to the leaf under consideration, *F_t_* is total LAI; *F/F_t_* is the relative LAI; *N_0_* and *N_a_* are leaf nitrogen concentration per unit leaf area at the top of the canopy (F = 0) and at *F* within the canopy, respectively; and *K_a_* is the coefficient of leaf N allocation [6], [7]. The attenuation of PPFD from uppermost leaves to lower leaves not only affects *N*_a_ distribution, but also alters chlorophyll composition, i.e., the chlorophyll a/b ratio, which decreases along with light attenuation in the rice canopy [8]. Leaf morphology varies along the light gradient. As an estimate of leaf thickness, leaf specific mass (the ratio of leaf dry weight to leaf area) also decreases from the upper to lower canopy [9].

Although PPFD plays a significant role in the formation of the *N*_a_ gradient in the canopy, other factors, primarily leaf age and N supply, are also important contributors [10]–[13]. Because lower leaves are physiologically older than upper ones, a gradient of *N*_a_ is formed along with the leaf age in the canopy. In a study of seasonal changes in leaf N in *Carex acutiformis*, the distribution pattern was found to be less uniform over the growing period [14]. Hikosaka et al. [10] used horizontally-grown vines of *Ipomoea tricolor* Cav. to evaluate the effect of leaf age on leaf N distribution separately from the effect of PPFD. Their study revealed a decreasing *N*_a_ gradient from the top to the base of the vine and the gradient became much steeper as plant age increased, though this effect was less pronounced in plants grown at higher nitrate concentrations. Wang et al. [15] found that differences in leaf N concentration between the fourth leaf and upper leaves (uppermost, second, and third) of rice (*Oryza sativa* L.) were reduced when plants were top-dressed with additional N. Explanations have unfortunately not yet been proposed for these changes in *N*_a_ distribution observed under different N treatments and during canopy development.

Determining leaf N or chlorophyll concentration which destroys subsamples is time-consuming and expensive. For example, each leaf was cut up and immediately placed in vials containing dimethyl sulfoxide solvent, chlorophyll extraction following described method by Hiscox and Israelstam [16]. Leaf N concentration was used to measure by the Kjeldahl method. However, leaf N concentration is closely correlated with chlorophyll content, which can be inferred from leaf color using a Soil-Plant Analysis Development (SPAD) chlorophyll meter. During the past decades, SPAD meters have facilitated researches on plant physiological ecology. Based on SPAD readings, leaf N and chlorophyll concentrations can thus be rapidly and nondestructively estimated [17]. Chang et al. [18] monitored changes in SPAD values in relation to growing degree days (GDD) in individual leaves. They observed three phases over the lifespan of a single leaf. During the first leaf growth phase, an exponential relationship was detected between leaf color and cumulative GDD. Relatively stable SPAD values were obtained during the leaf function period (the second phase). During leaf senescence (the third phase), the relationship between SPAD and GDD could be described by a quadratic equation. Inspired by this finding, we were motivated to explore *N*_a_ distribution by modeling SPAD value dynamics during the entire leaf lifespan.

There was a closely linear correlation between SPAD readings and *N_a_* about rice canopy leaves [19]. In this study, we used SPAD readings to examine *N*_a_ distribution in a rice plant canopy. The effects of leaf age, N supply, and light gradient on the distribution were investigated for two consecutive years in a paddy field. Because the PPFD gradient is altered when new leaves appear, it is generally difficult to distinguish its effect from those of leaf age and N rates. In our study, however, after all leaves fully expanded in rice plant, the rice canopy structure had little vary during the measurements, allowing us to assume constant canopy light distribution. The objectives of the present study were to model the dynamics of leaf SPAD readings over the leaf lifespan, develop a simulation model on leaf SPAD readings dynamic changes in rice canopy in relation to leaves age and elucidate effects of leaf age and N application rates on distribution changes of *N*_a_ (SPAD readings) in the rice canopy. In addition, we attempted to determine the pattern of gradient distribution of SPAD readings in rice canopy with increasing N application rates. Such results will be useful in maximizing photosynthesis in canopy leaves, improving the N fertilizer utilization efficiency of rice plants, and reducing environmental pollution. Meanwhile, SPAD readings replacing *N_a_* will provide a simple and convenient method to study the physiological and ecological characteristics of plants.

## Materials and Methods

### Ethics Statement

The experiments land is owned and managed by Hangzhou Academy of Agriculture, Hangzhou, China. Hangzhou Academy of Agriculture permits and approvals obtained for the work and study. The field studies did not involve endangered or protected species.

### Field Experiments

Field experiments were conducted in 2008 and 2009 on the experimental farm (120.09°E, 30.15°N) of the Hangzhou Academy of Agriculture, Hangzhou, China. Rice cultivar (*O. sativa*) “Bing 9363” were planted in a paddy field plot under six different N treatments using a randomized block design with three replicates. Seedlings with five or six fully expanded leaves were transplanted on June 27 of each year. Hill spacing was 0.23×0.13 m, with two seedlings per hill. A total of 608 hills were planted in each 3×6 m plot, resulting in a plant density of 67.6 plants m^−2^. Superphosphat (225 kg ha^−1^) and potassium chloride (75 kg ha^−1^) were incorporated into each plot on the day of transplantation; an additional 75 kg ha^−1^ of potassium chloride was applied as a top-dressing days after transplantation (DAT) 40 to prevent K deficiency. Plants received 0, 75, 150, 225, 300, or 375 kg N ha^−1^ in the form of urea, with each N rate applied in four doses based on rice growth stages as follows: July 3 (plant revival, 20%), July 10 (tillering, 30%), August 6 (panicle initiation, 30%), and August 30 (grain filling, 20%). The field consisted of loam paddy soil with organic matter and total N contents of 35.50 and 2.05 g kg^−1^, respectively.

### Measurement of SPAD Readings

At DAT 62 in 2008 and DAT 60 in 2009,all leaves of rice plants were fully expanded. A SPAD-502 chlorophyll meter (Minolta, Osaka, Japan) was used to obtain SPAD values (SPAD units) from the four uppermost fully expanded leaves on each plant over approximately 7-d intervals (depending on weather). Ten plants were measured in every plot. Three SPAD readings were obtained per leaf, one near the leaf blade midpoint and the other two located 3 cm to either side of the midpoint, and averaged as the mean SPAD reading of the leaf [17]. To observe temporal dynamics of SPAD readings at the same leaf position under different N rates, we labeled fourteen leaves (2009) (all leaves were numbered acropetally) in each plot and obtained SPAD readings immediately after they fully expanded (i.e., the blade stopped elongating). The measure method was the same as above. To more accurately estimate N concentration, a leaf SPAD reading was calculated by taking the mean of 10 SPAD readings from leaf base to apex. Measurement intervals were 2 or 3 d in 2009.

### Leaf Area Determination

Azimuth angles of the leaves were supposed to distribute uniformly. The LAI of leaf was obtained by summing the area for each leaf and each inclination angle class. To calculate cumulative LAI of the four uppermost leaves, 10 tillers from each plot were chosen and the leaf areas of their four uppermost leaves were measured using an AM100 leaf area meter (ADC, UK). The average leaf area (ALA) of the uppermost leaves was then calculated. In addition, the number of tillers observed in 10 hills of each plot was counted and the average tiller number (ATN) per plot calculated. The cumulative LAI of uppermost leaves was calculated as follows: 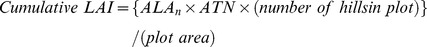
(2)


Where *ALA_n_* is the average total leaf area of the uppermost *n* leaves on the same tiller (*n*  =  1, 2, 3, or 4).

### Measurement of Chlorophyll Concentration and N Concentration

Immediately after the last leaf had fully expanded, 20 leaves were obtained from each plot. Ten of the collected leaves were extracted using 96% (v/v) ethanol, and their chlorophyll concentrations were determined by measuring absorbance at 649 and 665 nm using a Lambda 45 spectrophotometer (Perkin-Elmer, USA). The remaining leaves were dried at 70°C until a constant weight, and leaf N concentrations were then determined by the Dumas combustion method [20] on a Rapid N Cube nitrogen analyzer (Elementar, Germany). Leaf N and chlorophyll concentrations were measured four times at 10-d intervals.

### Measurement of PPFD and morphological and biochemical characteristics

Light intensity at each canopy position was measured using an external quantum sensor of a Li-6400 gas analyzer (LI-COR, USA). All canopy leaves of rice plants were fully expanded at the time of measurement. The quantum sensor was held in the same angle as the leaf. The measurements were taken from the four uppermost fully expanded leaves on each plant at approximately 6-d intervals. PPFD was read 20 times for every leaf and the mean calculated. To explore the morphological and biochemical characteristics of leaves at different canopy positions, 20 tillers were selected on September 10, 2008 from one plot without N application. Leaf area, thickness, and SPAD readings were determined for the top four leaves of each rice tiller. Leaf thickness was measured using a displacement sensor [21]. N and chlorophyll concentrations were determined as described above. To examine the details of upper and lower leaf morphological differences under varied light intensities, leaves at different positions were removed from the above-mentioned plot; leaf cross sections were then stained and photographed [22].

### Development of a Canopy Leaf SPAD Readings Model

The steepness of the gradient of SPAD values was evaluated in terms of *K_SPAD_*, a coefficient of leaf SPAD reading distribution calculated according to the formula:
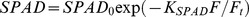
(3)


Where *F* and *F_t_* denote LAI cumulated from the uppermost leaf to the 4th leaf and total cumulative LAI, *SPAD_0_* is the SPAD reading of the uppermost leaf and *SPAD* is the reading at the canopy leaf position *F*. When SPAD readings are uniformly distributed in the canopy, *K_SPAD_*  =  0; values increase from uppermost to lowermost leaves if *K_SPAD_* < 0, and decrease if *K_SPAD_* > 0.

### Data Analysis

Data for each sampling date and year were subjected to analyses of variance using SPSS16.0 (Chicago, IL, USA) and the LSD test was used to assess differences between treatment means. The linear and quadratic regression analysis was performed between SPAD readings and DAT, *N_a_* using SPSS 16.0 (Chicago, IL, USA). Intercepts and slopes of regression curve at *different* developmental stages were compared using the model procedures of SPSS 16.0 (Chicago, IL, USA) and Origin 8.0.

## Results

**Figure 1:**
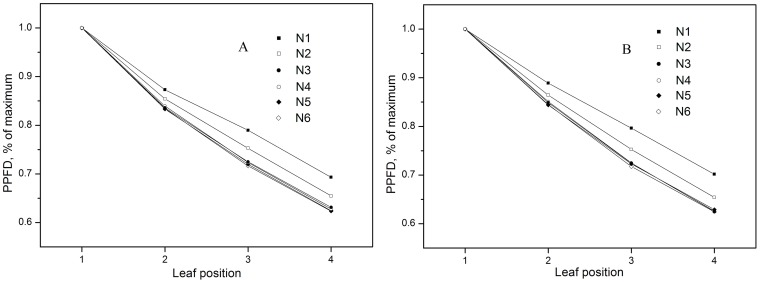
Photosynthetic photon flux density (PPFD) in relation to canopy position. Data are represented in terms of the percentage of PPFD measured at the uppermost canopy position. Measurements were taken at midday at the flag leaf and the next three leaves (2nd, 3rd, and 4th leaves) on (A) Sept. 1, 2008, and (B) Aug. 26, 2009. N1, N2, N3, N4, N5, and N6 indicate N application rates of 0, 75, 150, 225, 300, and 375 kg N ha^−1^, respectively.

Light gradients were formed in the rice canopy and increased in steepness as more N was applied (Fig. 1). In both years, some leaf physiological and biochemical characteristics, such as chlorophyll concentration, chlorophyll a/b ratio, *N_a_*, specific leaf mass, and leaf thickness, significantly decreased from uppermost to lowermost leaves along the light gradient (Table 1, P< 0.05).

**Table 1:** Chlorophyll concentration (g m^−2^), chlorophyll a/b ratio, N concentration (*N*_a_, g m^−2^), and leaf thickness (µm) of the four uppermost leaves of rice plants from three plots not subjected to N application.

Leaf Position	Chl concentration (g m^−2^)		Chl a/b ratio		N_a_ (g m^−2^)		Leaf thickness(µm)	
	2008	2009	2008	2009	2008	2009	2008	2009
1st	0.23±0.020a	0.25±0.017a	2.66±0.12a	2.71±0.17a	1.10±0.09a	1.12±0.12a	213±16.2a	196±14.9a
2nd	0.20±0.023b	0.23±0.023b	2.42±0.14b	2.56±0.13b	0.84±0.15b	0.87±0.10b	182±13.5b	172±13.7b
3rd	0.17±0.034c	0.19±0.031c	2.33±0.13c	2.32±0.15c	0.60±0.13c	0.65±0.15c	163±14.2c	154±15.2c
4th	0.15±0.038d	0.13±0.028d	2.38±0.12c	2.29±0.14c	0.52±0.14d	0.41±0.17d	158±12.8d	145±13.5d

Each value is an average of 30 measurements. Within a column, values followed by different letters are significantly different at *P* <0.05 according to Duncan’s multiple range test. Dates of sampling were DAT 75 in 2008 and DAT 77 in 2009.

Canopy structure was little changed during the study period, as no additional leaves appeared after DAT 62 in 2008 or DAT 60 in 2009, and no lower leaves wilted. On August 24 of both years, the last (uppermost) leaf of the plant was fully expanded; the canopy structure and cumulative LAI of the top four leaves consequently remained constant in the field until approximately October 10, when the fourth leaf started to wither away as a result of low N supply. As a consequence, canopy light gradients remained constant. Approximately 15 leaves were present on main stem tillers. It took 6 – 8 d for the final four leaves to fully develop following emergence during the rice reproductive stage. Consequently, for the last four leaves, the lower leaf was physiologically 6 – 8 d older than the leaf immediately above it, which created a “leaf age difference” (leaf age difference  =  lower leaf age – upper leaf age) between the two adjacent leaves. As a result, a leaf age gradient was generated along the tiller.

**Figure 2:**
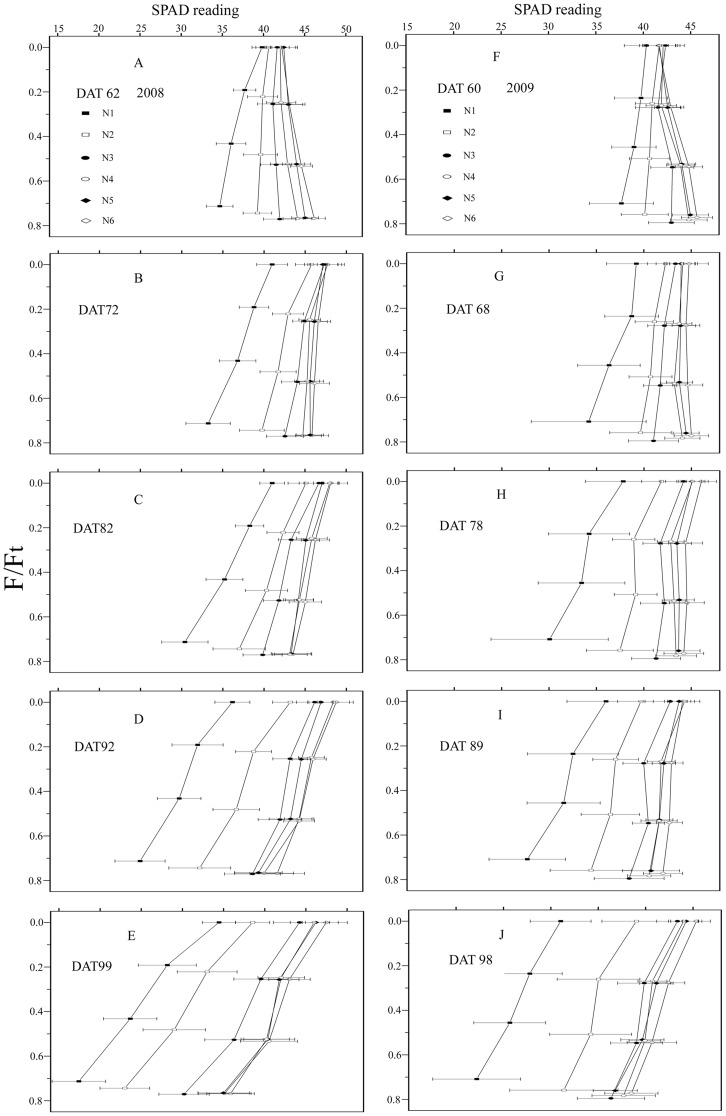
Temporal changes in the distribution of SPAD readings of rice plants grown under six N application rates during (A - E) 2008 and (F - J) 2009. *F* is the cumulative leaf area index (LAI) from the top of the canopy, *F_t_* is total LAI; *F/F_t_* is the relative LAI. Each value is an average of 30 measurements. Bars indicate standard error of means. N1 to N6 are the same as in the legend in Fig. 1.

**Figure 3:**
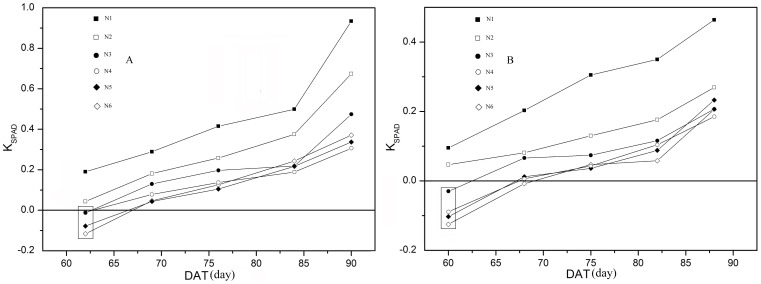
Temporal dynamics of K_SPAD_ in plants grown under different N application rates. K_SPAD_ values were calculated from the data in Fig. 3. Data in the boxes are negative, indicating that SPAD readings increased with increasing canopy depth. N1 to N6 are the same as in the legend in Fig. 1.

**Figure 4:**
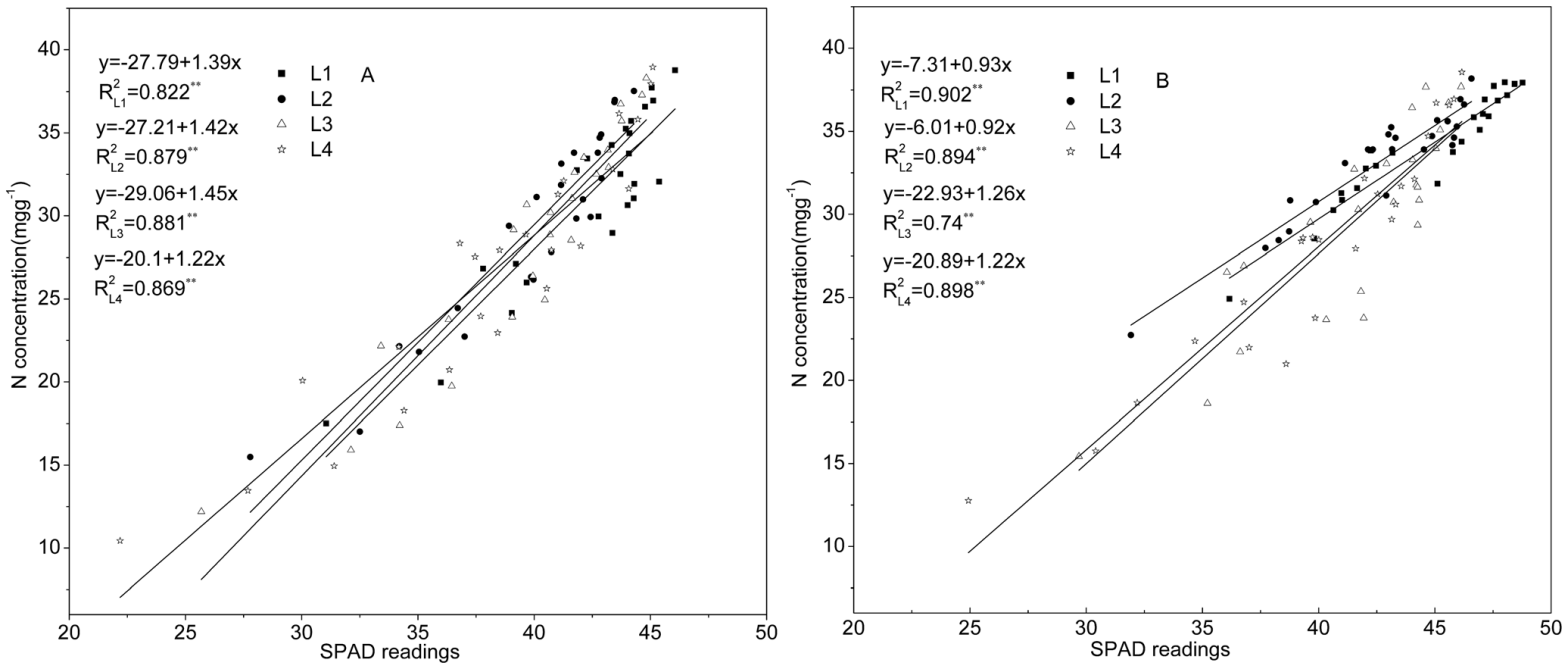
Linear regression relationship between SPAD readings and *N_a_* in the canopy leaves after DAT 62 in (A) 2008 and DAT 60 in (B) 2009. L1, L2, L3 and L4 represent the first, second, third and fourth fully expanded leaf from the top of plant, respectively. L_pool_ represent pooled datum of L1 - L4 SPAD readings. Solid lines represent linear regressions.

Figure 2 illustrates the distribution of leaf SPAD values over time in rice canopies subjected to six different N application rates during the 2 years of the study. The distributions of SPAD readings were compared in terms of *F/F_t_*. The data at *F/F_t_  = * 0 represent SPAD readings of the uppermost leaves. The dynamic changes of SPAD readings for five periods during 2008 and 2009 are shown in Fig. 2A - E and F - J, respectively. For each period and N application rate, the power function [equation (3)] provided a significant fit to the *SPAD* vs. *F/F_t_* data (*P* <0.01). As might be expected, SPAD readings usually decreased from the top to the bottom of the canopy (Fig. 2, B - E, G - J), although the reverse trend was observed in a few cases (Fig. 2A and F). On DAT 62 of 2008 and DAT 60 of 2009, when the uppermost leaves were newly expanded, leaf SPAD readings of rice plants subjected to pure N levels of 150 to 375 kg ha^−1^ increased from top to bottom, as indicated by the negative *K_SPAD_* shown in Fig. 3. In these cases, the distributional gradient increased in steepness with increasing N application rates. With the aging of plant, however, this atypical distribution of canopy leaf SPAD readings quickly reversed to the usual order (Figs. 2 and 3). Except for these sporadic negative *K_SPAD_* values, however, values progressively increased over time, indicating that SPAD readings distributional gradients gradually became steeper as plant development progressed (Fig. 2). In addition, *K_SPAD_* was much lower, and hence gradients tended to be less steep, when plants were top-dressed with higher levels of N (Figs. 2 and 3). Consequently, the distribution of leaf SPAD readings is significantly affected by nitrogen availability, with differences between upper and lower leaves decreasing with increasing N applications. Linear regression relationship between SPAD readings and *N_a_* is very close in the canopy leaves after DAT 62 in (A) 2008 and DAT 60 in (B) 2009 (Fig. 4). This result is similar to Esfahani et al [19].

**Figure 5:**
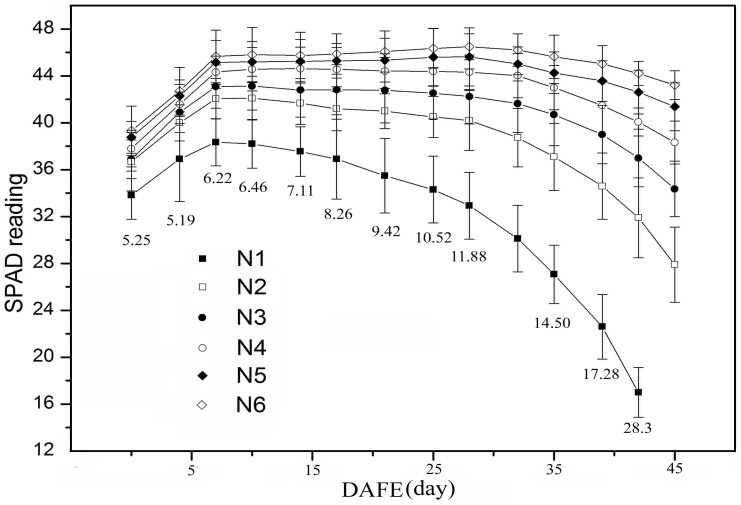
Temporal dynamics of SPAD readings of labeled rice leaves under six N application rates. Each value is an average of 30 measurements. Bars indicate standard error of means. Numbers underneath bars are coefficients of variation (%) of SPAD readings obtained on the same day under the six N application rates. SPAD readings are not shown for control plants on the 45th day after full expansion (DAFE 45) because some leaves in plots without N application were dead. N1 to N6 are the same as in the legend in Fig. 1.

**Figure 6:**
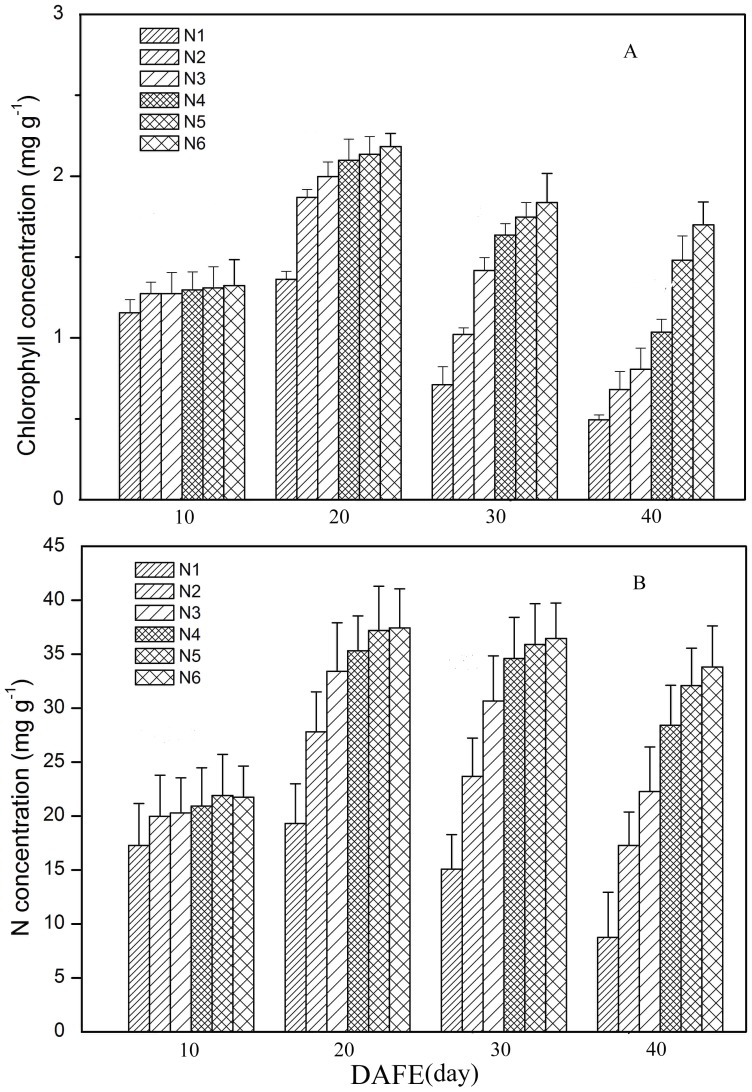
Temporal dynamics of chlorophyll concentration (based on per - unit fresh weight) and N concentration (based on per - unit dry weight) of individual leaves under six N application rates. N1 to N6 are the same as in the legend in Fig. 1.

Changes in rice leaf SPAD readings over leaf lifespan varied markedly among different N application rates (Fig. 5). To clearly elaborate the dynamics of leaf SPAD readings, leaf lifespan was divided into three stages, namely leaf growth, function, and senescence, following Chang et al. [17]. Actually, the division of the physiological function in the leaf life span was not so distinct and is only to facilitate describing the dynamics of leaf SPAD readings. Leaf growth periods under different N application rates were brief, all lasting approximately 6 d. The leaf function period was prolonged by the application of N, and SPAD readings during this period were also elevated (Fig. 5). In plants not subjected to N application, the leaf function period was very short (less than 3 d), whereas it lasted 20 d in plants applied with 375 kg ha^−1^ N (Fig. 5). In addition, leaf senescence, assessed by the decrease in SPAD readings, was slowed at higher N levels. At each measured time point, the space for SPAD readings among the six N application rates increased as leaves aged, implying that the differences in leaf SPAD readings under different N application rates were magnified with leaf development (Fig. 5). Similar to the SAPD readings, the difference for chlorophyll and N concentrations of uppermost leaves under different N application rates also increased with leaf age (Fig. 6). At DAT 60 in 2009, immediately following the full expansion of uppermost leaves, leaf chlorophyll and N concentrations did not vary much between different application rates, but the difference become more distinct as the leaves aged.

**Figure 7:**
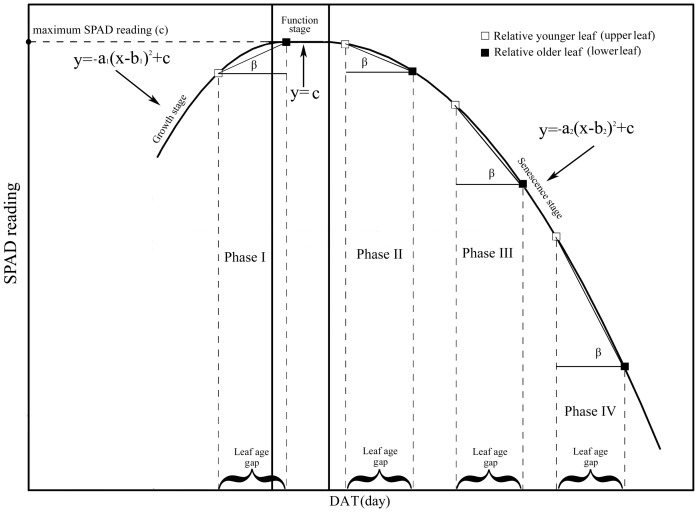
Temporal dynamics of SPAD readings over leaf lifespan fitted to a piecewise function. Leaf lifespan was divided into three growth stages: growth, function, and senescence. The leaf age difference is the difference in leaf physiological age between upper and lower leaves. The dynamics of leaf SPAD readings of upper and lower leaves were assumed to follow the same trajectory (piecewise function). Angle *β* served as an index of the SPAD reading distribution gradient.

SPAD readings over leaf lifespan under different N application rates were fitted to a piecewise function (Fig. 7). During the leaf function period the SPAD readings were relatively stable. Results of the function fitting are shown in Table 2. During the leaf senescence, we found the rate of decrease in SPAD readings with advancing leaf age was evaluated as:

**Table 2:** Results from the fitting of dynamics of SPAD readings to a piecewise function.

N application rate	Growth stage			Function stage		Senescence stage		
	a_1_	R^2^	n	c	n	a_2_	R^2^	n
N1	0.041***	1	3	37.6	1	0.024	0.991***	9
N2	0.021***	1	3	38.2	2	0.017	0.979***	9
N3	0.039***	1	3	42.9	3	0.012	0.970***	8
N4	0.006***	1	3	44.6	4	0.010	0.972***	7
N5	–0.010***	1	3	45.3	4	0.008	0.995***	7
N6	–0.020***	1	3	46.0	5	0.009	0.983***	6

The letters a_1_, c, and a_2_ represent function coefficients as shown in Fig. 6. N1, N2, N3, N4, N5, and N6 indicate N application rates of 0, 75, 150, 225, 300, and 375 kg N ha^−1^, respectively.

^***^ *P* <0.001.



**Figure 8:**
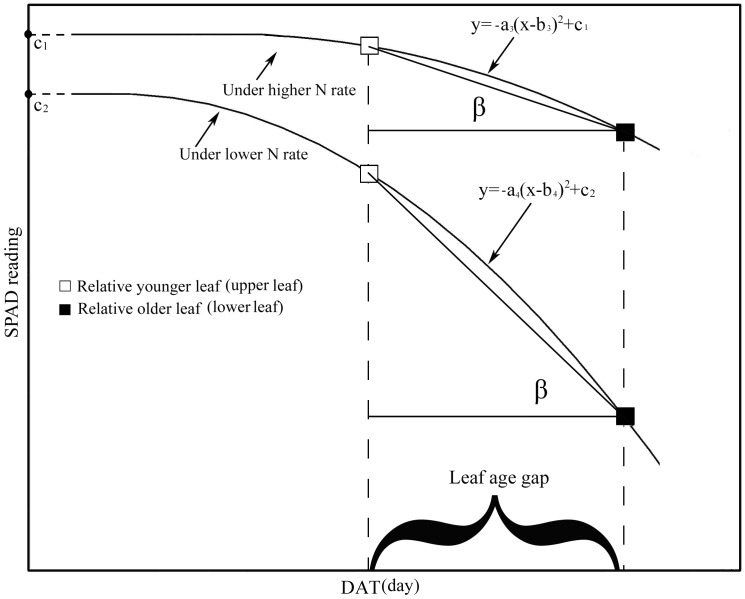
Temporal dynamics of SPAD readings over leaf lifespan under higher and lower N rates fitted to a piecewise function during leaf growth and senescence stages. Dynamics of upper and lower leaves were assumed to follow the same trajectory (piecewise function).

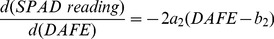
(4)Where *a_2_* is the absolute value of the quadratic coefficient, *DAFE* is the number of days following leaf full expansion, and *b_2_* is the DAFE when SPAD readings indicated onset of senescence. As revealed by equation (4), the rate increased as *DAFE* (leaf age) increased. During the growth and senescence stages, a quadratic relationship was observed between SPAD readings and DAT. The absolute value of the quadratic coefficient was elevated by the N application, indicating that, during leaf senescence, the rate of decrease in SPAD readings was slowed by N application (Table 2 and Fig. 8). As can be seen in Fig. 8, *a_3_*, the absolute value of the quadratic coefficient under higher N application rates, is clearly smaller than *a_4_*, the value under lower N application rates.

## Discussion

The much steeper light gradient observed in canopies under higher N application rates can be attributed to the droopier leaves obtained at these rates [23]. Leaves oriented more horizontally tend to intercept more light, resulting in less illumination for the lower leaves. Leaf physiological and biochemical characteristics differed greatly among the different leaf positions (Table 1), a reflection of acclimation to the light gradient in the canopy [8]. Leaf characteristics and irradiance experienced by the leaves are closely related. Leaves grown under higher irradiance are characterized by greater leaf thickness, higher chlorophyll a/b ratios, and higher chlorophyll and *N_a_* [24], [25]. Most importantly, in our study, the light gradient affected the distribution of leaf SPAD readings (chlorophyll and N concentrations) in the canopy.

Because the canopy in the paddy field had little change during the measurement period (after all the leaves of the rice plant had fully expanded and before they began to wither and fall), canopy light gradients under the same N treatment remained constant over the course of the study. The temporal dynamics of SPAD readings under each N application rate can therefore be attributed primarily to increasing plant age. This finding is consistent with those of previous studies [8], [26]. Furthermore, while light gradients in canopies treated with higher N levels were much steeper (Fig. 1), SPAD readings in these canopies comprised a smaller range of values (Fig. 2), confirming that N application induce a flatter SPAD reading distribution. This phenomenon is consistent with Dreccer et al [27].Changes in leaf chlorophyll and N concentration over leaf lifespan should be correlated with leaf SPAD readings. This accounts for the gradual increase observed in difference of chlorophyll and N concentrations under six N application rates with advancing leaf age (Fig. 6). In addition, because the area of fully expanded leaves is fixed, the dynamics of chlorophyll and N concentration with respect to area were the same as those expressed on per-unit fresh or dry weight basis (Fig. 6). Based on SPAD dynamics of individual leaves, we can propose an explanation for the above-mentioned observations. We first assume that the temporal dynamics of upper and lower leaves on the same tiller, which was subjected to the same N application rate, follow a similar trajectory during senescence (Fig. 7). As mentioned above, a leaf age difference existed between the upper and lower leaves, resulting in the distribution of SPAD readings on upper (younger) and lower (older) leaves. As illustrated in Fig. 7, the steepness of the SPAD reading distribution is represented by the angle *β*. When the upper leaf begins to senesce (canopy in phase II), a SPAD reading gradient is formed. As the canopy develops during phases III and IV, the gradient becomes much steeper. This is reflected by the increase in *β* with DAT, showing the characteristics of a quadratic function (Fig. 7). To summarize, the continuously accelerating rate of chlorophyll or N degradation in lower (older) leaves is the factor responsible for the increased difference in SPAD readings between upper (younger) and lower leaves, which in turn brings about the gradually steeper SPAD reading distribution as the canopy develops. The assumption made in the first step, however, may not be exactly correct. Chlorophyll degradation is regulated by phytochrome [28], and it has been reported that potato (*Solanum tuberosum* L.) leaves rapidly senesce when subjected to extreme shade [29]. This suggests that leaf senescence is accelerated in heavy shade [28]. If this is the case in the rice canopy, differences between SPAD readings from upper (less shaded) and lower (more shaded) leaves will be further increased. In the cited studies, however, senescence was accelerated by imposition of darkness or dim light, which are unnatural treatments. The role of light in the control of senescence under natural conditions is not clear.

A similar interpretation can likewise be made for the less steep gradient of SPAD readings observed under higher N application rates (Fig. 8). During leaf senescence, the rapid drop in leaf SPAD readings is suppressed in plants subjected to higher N application rates, which is reflected by the lower absolute values of the quadratic coefficient shown in Table 2. As a result, using the same assumption made above, the difference between SPAD readings from upper and lower leaves is reduced when higher levels of N are applied (Fig. 8). Viewed from another perspective, the distribution of *N*_a_, which is affected by N supply, may be due to the remobilization of N from old leaves to young leaves [30], [31]. Mae and Ohira [30] determined that leaf blades are the primary source of remobilized N with at least half of the total N in growing tissues derived from remobilized N. Lower leaves are therefore important N suppliers for their upper leaves in their younger growth stage. When upper leaves become deficient in N, more N will be translocated from the lower leaves. Thus, the difference in leaf N concentrations between the upper and lower leaves will increase if a crop is under N stress.

On two occasions, DAT 62 in 2008 and DAT 60 in 2009, leaf SPAD readings of plants subjected to 150 to 375 kg N ha^−1^ increased with increasing canopy depth. These results may be due to the obvious prolongation of the plant function stage arising from higher N application rates (Fig. 5 and Table 2). While the upper leaves were in the growth stage, the lower leaves were still experiencing the prolonged function stage (phase I in Fig. 7). With canopy development, the lower (older) leaves entered senescence; as a result, leaf SPAD readings in the canopy gradually became more uniform (*K_SPAD_* close to 0) followed by a distribution in the reverse direction (*K_SPAD_* > 0) (Fig. 3). K_SPAD_ < 0 mean lower leaves having higher SPAD readings. Because N fertilizer was excessively applied in paddy fields (N5 or N6), the N concentration of lower leaves was close to the upper leaves, even higher [15]. Similarly, the lower leaves SPAD readings would be higher under the condition of excessive N rate.

It should be reemphasized that the assumption used in the above interpretations is not completely incontrovertible. In addition to N application rate, there may be numerous other factors that affect the coefficients of the equation describing temporal changes in leaf SPAD readings. For example, during the reproductive stage, canopy leaves serve as a resource, supplying photosynthate and N to rice grains. In sunflower (*Helianthus annuus* L.), N is mobilized from leaves in all positions; the highest rate of change in N is observed in leaves closest to the grain, where they can facilitate the supply of N to the highly-demanding process of growing grain [32]. Whether this is the case for the last four leaves in the rice canopy, consequently affecting the rate of decrease in SPAD readings, is uncertain and requires further investigation. In addition to the difference in shade degree mentioned above, rates of decrease in SPAD readings in different leaf positions may also be more or less influenced by environmental factors such as ambient temperature and humidity. Of all these factors, however, N application rate is likely the primary factor influencing the rate of decrease.

Regardless of complicated factors external or internal to the plants, the dynamics of leaf SPAD readings at different leaf positions could be modeled by three coefficients of the fitted equation: *a_2_*, *DAFE*, and *b_2_* [equation (4)]. Consequently, the temporal distribution of canopy SPAD readings under different N application rates can be attributed to two factors: (1) the influence of N application rate (and possibly other factors, including shade) on *a_2_* and *b_2_*; and (2) leaf age differences between upper and lower leaves (much larger *DAFE* for lower leaves compared with upper leaves). These two factors give rise to the observed variation in the rate of decreased SPAD readings (*N_a_*). The effects of leaf age and N application on SPAD reading (*N*_a_) distribution can be ascertained through mathematical analysis of these two factors. Even our hypothesized situation is overly simplified. A similar conclusion might be drawn by the consideration of additional factors. In conclusion, by analyzing leaf SPAD reading (*N*_a_) dynamics at different leaf positions, we have provided insights into plant canopy *N*_a_ distribution.
