# Correction: Effects of Nitrogen Application Rate and Leaf Age on the Distribution Pattern of Leaf SPAD Readings in the Rice Canopy

**DOI:** 10.1371/journal.pone.0092509

**Published:** 2014-03-10

**Authors:** The PLOS ONE Staff

The number of a grant from the National Natural Science Foundation of China is incorrect. The Funding statement should read: “The research was financially supported by National Natural Science Foundation of China (No. 60574046, 61174089) and Zhejiang Natural Science Foundation (No. Y507005). The funders had no role in study design, data collection and analysis, decision to publish, or preparation of the manuscript.” The publisher apologizes for this error.
